# Pregnancy decisions and contraceptive use among HIV-positive women: a study in a large urban clinic in sub-Saharan Africa

**DOI:** 10.1186/1758-2652-13-S4-P164

**Published:** 2010-11-08

**Authors:** J Walusimbi, E Birabwa, E Nabankema, A AnneMarie, I Lutalo, A Nakiwooga

**Affiliations:** 1 Infectious Diseases Institute, Prevention, Care and Treatment, Kampala, Uganda; 2 Infectious Diseases Institute, Monitoring and Evaluation, Kampala, Uganda

## Background

The IDI is an HIV treatment and research centre in Kampala, Uganda with over 24000 patients of whom 9000 are on antiretroviral therapy.

## Purpose of the study

We conducted a study in March 2007 to determine the accessibility and utilization of Sexual Reproductive Health (SRH) services among female clients.

## Methods

Using a structured questionnaire, a cross-sectional survey of female clients aged 18-49 years attending the IDI clinic was conducted. SPSS version 12.0 was used to fit a logistic regression model to determine the following outcomes; pregnancy decisions, desire for children and pregnancy risk behaviour among sexually active female clients.

## Results

Of 493 respondents, 322 (65%) were sexually active at the time of the survey. Over 30% of the respondents had become pregnant after knowing their sero-status, 66% of the pregnancies were unintended of which 39% ended in abortions. Over 52% of the pregnancies were due to the influence of the husband, 33% was a result of mutual agreement between the clients and their partners while 15% of them were because of the client's decision. Of the women who made their own decision about pregnancy, 57% had a secondary level of education. Among married 40% of the pregnancies were a result of mutual agreement while relatives influenced 45% of the pregnancies among the singles. Of the participants 96% reported awareness of family planning methods; however, the level of utilization was at 40%. Overall 31% of the women stated a desire for children. 41% engaged in pregnancy risk behaviour and of these 63% did not desire children. Women aged 24-34 years had the highest desire to have children. The husbands made pregnancy decisions for 62% of the women who did not want more children.

## Conclusions

Family planning utilization is low even among those females who have no desire for more children resulting in unwanted pregnancies. Despite their HIV status women remain sexually active and have a desire for more children. A level of education had no bearing on contraceptive use but was important for decision making about pregnancy.

